# Changes in Metabolomics Profiles of *Propylea japonica* in Response to Acute Heat Stress

**DOI:** 10.3390/ijms26104541

**Published:** 2025-05-09

**Authors:** Yang Xu, Lishan Diao, Xiaojie Yang, Man Zhao, Yuqiang Xi, Yanmin Liu, Weizheng Li, Gaoping Wang, Meiling Fang, Xianru Guo, Lijuan Zhang

**Affiliations:** College of Plant Protection, Henan Agricultural University, Zhengzhou 450046, China; xy2320370189@163.com (Y.X.); diaolshan@163.com (L.D.); xiaojieyang923@163.com (X.Y.); zhaoman821@126.com (M.Z.); yuqiangxi2012@126.com (Y.X.); lym09b1@163.com (Y.L.); wei-zhengli@163.com (W.L.); cnhnzzwang@126.com (G.W.); fml6218@163.com (M.F.)

**Keywords:** *Propylea japonica*, metabolomics, thermotolerance, energy metabolism, membrane homeostasis

## Abstract

The ladybird beetle, *Propylea japonica* Thunberg (Coleoptera: Coccinellidae), is a widely distributed natural predator that is crucial in controlling various agricultural pests in China. Despite frequent references to its remarkable thermotolerance, the molecular mechanisms underlying its thermotolerance remain poorly understood. Here, we investigated metabolomic changes in *P. japonica* following exposure to acute heat stress (AHS) lasting 1 h at 39 °C and 43 °C in populations from Zhengzhou (ZZ, warm temperate climate zone) and Shenzhen (SZ, subtropical climate zone), representing distinct northern and southern Chinese ecosystems. A total of 4165 and 4151 metabolites were detected in positive and negative ion modes, respectively. The high proportion of lipid and lipid-like metabolites (35.5%) and the top 20 pathways containing the highest number of metabolites, implying membrane fluidity modulation and energy metabolism **restructuring,** served as the core adaptive mechanism in *P. japonica* populations confronting thermal stress. The SZ25 vs. SZ39 exhibited a significantly higher number of differentially expressed metabolites (DEMs), which were predominantly enriched in the purine and tryptophan metabolism pathways. This indicated that these pathways orchestrate thermal adaptation in the SZ population by coordinating energy metabolism reprogramming, orchestrating antioxidant defense mechanisms, and modulating neuroendocrine homeostasis dysregulation. Additionally, the starch and sucrose, arachidonic acid, and fructose and mannose metabolism pathways were also implicated. This study enhances our understanding of *P. japonica* thermotolerance and provides a valuable reference for thermotolerance mechanisms in other insects.

## 1. Introduction

Terrestrial insects experience various environmental stressors, with temperature being a critical abiotic factor. As ectotherms, insects are highly sensitive to temperature fluctuations. For instance, leaf-cutting ants (*Atta vollenweideri*) can detect temperature changes as small as 0.005 K [1]. However, with global temperatures rising and extreme temperature events becoming more frequent [2], insects are expected to face intensified thermal stress. Temperature fluctuations impact numerous biological processes, including behavior, reproduction, and physiology [3,4,5]. For example, temperature influences flight performance in damselflies (*Mnais costalis*) [6], and mating rates in the spotted wing *Drosophila* (*Drosophila suzukii*) drop sharply outside the optimal range of 15–30 °C [7]. In *Epiverta chelonia*, a ladybird species, egg hatching is nearly impossible at temperatures of 28 °C or higher [8]. Furthermore, exposure to extreme heat (48 °C and 50 °C) severely disrupts the potassium ion balance in the migratory locust (*Locusta migratoria*) [9].

As a key predatory insect, *P. japonica* plays a significant role in biological pest control, preying on aphids, planthoppers, whiteflies, spider mites, and the eggs and larvae of certain lepidopterans [10,11,12]. Native to Asia [12], *P. japonica* is widely distributed across China. Extensive research has explored various aspects of its biology, including the assembly of a chromosome-level genome in 2019 [13], as well as studies on its morphological diversity (color spot variation), life history, high reproductive capacity, and ecological adaptations, such as strong resistance to insecticides and high temperatures [13,14,15,16]. Given ongoing climate warming, the thermotolerance of *P. japonica* will become increasingly important. Previous studies have shown that exposure to 36 °C negatively affects its development across multiple life stages and significantly skews the adult sex ratio towards males [17]. At 43 °C, mass mortality occurs [18,19]. So, physiological responses to heat were assessed in this species.

Metabolomics is a powerful tool for investigating physiological responses by profiling low-molecular-weight endogenous metabolites (<1000 Da) and capturing biochemical shifts under stress conditions [20,21,22]. Rapid advancements in this field have expanded its applications across disciplines, including human disease research, pharmaceuticals, and agriculture [23,24,25]. In this study, we employed untargeted ultra-performance liquid chromatography-tandem mass spectrometry (UPLC-MS/MS)-based metabolomics to analyze the metabolic responses of *P. japonica* to AHS. Considering the maximization of temperature variation within the species’ suitable habitat range, we strategically sampled sites spanning climate zone gradients from ZZ to SZ, thereby capturing thermal regimes representative of both warm temperate semi-humid zones and subtropical monsoon regions. Two treatment temperatures (39 °C and 43 °C) were selected based on preliminary thermal tolerance thresholds and quantitative shifts in insect-derived bioactive components. This study aims to elucidate the key metabolic determinants and regulatory pathways underlying thermotolerance mechanisms and decipher how *P. japonica* drives adaptive responses through the regulation of key metabolites and metabolic pathways under elevated temperature conditions. Finally, this study will provide foundational data for the targeted modulation of key metabolic pathways and the breeding of heat-tolerant strains.

## 2. Results

### 2.1. Data Quality Assessment

**Figure 1:**
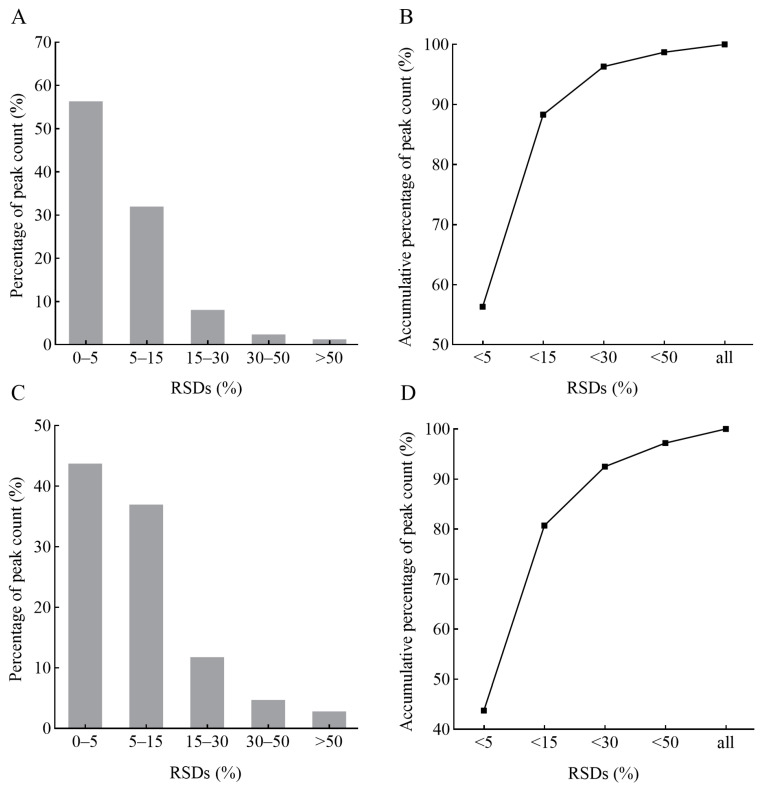
Distribution pattern of RSDs for detected peaks. Bar plots and line charts represent peak distributions in positive ion mode (**A**,**B**) and negative ion mode (**C**,**D**). The *x*-axis shows different RSD (%) ranges, while the y-axis represents the proportion of peaks.

In this study, we analyzed the metabolomic profiles of *P. japonica* using UPLC-MS/MS with relative quantification. A total of 108 samples (six experimental groups × three biological replicates × six individuals) were analyzed. Additionally, four quality control (QC) samples (QC-1, QC-2, QC-3, and QC-4) were included to assess data reliability. Data quality was evaluated using Spearman’s correlation coefficients between QC samples and the relative standard deviations (RSDs) of peak abundance values. The results indicated that correlation coefficients between any two QC samples exceeded 0.983 in positive ion mode (Table S1) and 0.965 in negative ion mode (Table S2). Furthermore, 96.33% of peaks in positive ion mode (Figure 1A,B) and 92.48% in negative ion mode (Figure 1C,D) had RSDs below 30%, demonstrating the high reproducibility of QC samples and the stability of the analytical platform. These findings confirm that the acquired data were reliable for subsequent analyses.

### 2.2. Overview of Metabolomic Profiles

**Figure 2:**
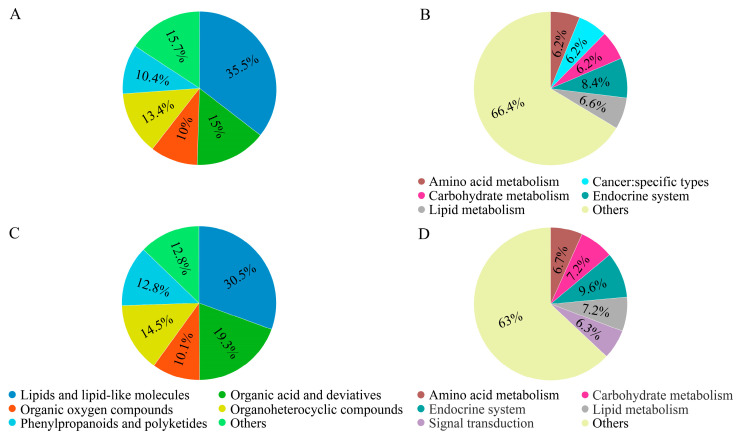
Pie charts depict the classification of identified metabolites in positive ion mode (**A**); enriched KEGG pathways in positive ion mode (**B**); classification of identified metabolites in negative ion mode (**C**); enriched KEGG pathways in negative ion mode (**D**).

Metabolomic changes in *P. japonica* under AHS were analyzed using UPLC-MS/MS. In positive ion mode, 18,661 peaks were detected, identifying 4167 metabolites. These were primarily classified into lipids and lipid-like molecules (35.5%), organic acids and derivatives (15%), organoheterocyclic compounds (13.4%), and phenylpropanoids and polyketides (10.4%) (Figure 2A). The synergistic interactions of lipids, organic acids, and antioxidant metabolites may establish a ‘thermotolerance metabolic module’, aiding *P. japonica* in maintaining cellular homeostasis. The high proportion of lipids and lipid-like molecules (35.5%) suggests that membrane restructuring is a central strategy for the *P. japonica* population to adapt to high temperatures, while the presence of phenylpropanoids/polyketides likely reflects its dual adaptation to tropical environments with intense UV radiation and thermal stress. KEGG annotation mapped 1183 metabolites, with 564 involved in 226 metabolic pathways. The most enriched pathways included the endocrine system (8.4%), lipid metabolism (6.6%), amino acid metabolism (6.2%), and carbohydrate metabolism (6.2%) (Figure 2B). The higher enrichment of lipid metabolism (6.6%) suggests that the remodeling of membrane systems is a core strategy for *P. japonica* thermotolerance, while the highest proportion of the endocrine system (8.4%) likely reflects its dominant role in cross-pathway regulation.

In negative ion mode, 19,806 peaks were detected, identifying 4155 metabolites. The major categories included lipids and lipid-like molecules (30.5%), organic acids and derivatives (19.3%), organoheterocyclic compounds (14.5%), and phenylpropanoids and polyketides (12.8%) (Figure 2C). A total of 1066 metabolites were mapped to the KEGG database, with 580 involved in 208 metabolic pathways. Key enriched pathways included the endocrine system (9.6%), carbohydrate metabolism (7.2%), lipid metabolism (7.2%), amino acid metabolism (6.7%), and signal transduction (6.3%) (Figure 2D). In a word, the results implicated that the endocrine system acts as a ‘central regulatory hub’, coordinating the metabolic reprogramming of lipids, amino acids, and carbohydrates to orchestrate multi-layered thermotolerance responses. For a short-term response, rapid carbohydrate breakdown provides immediate protection against heat stress. For long-term adaptation, lipid remodeling and sustained amino acid metabolism support the development of a stable thermotolerant phenotype.

**Figure 3:**
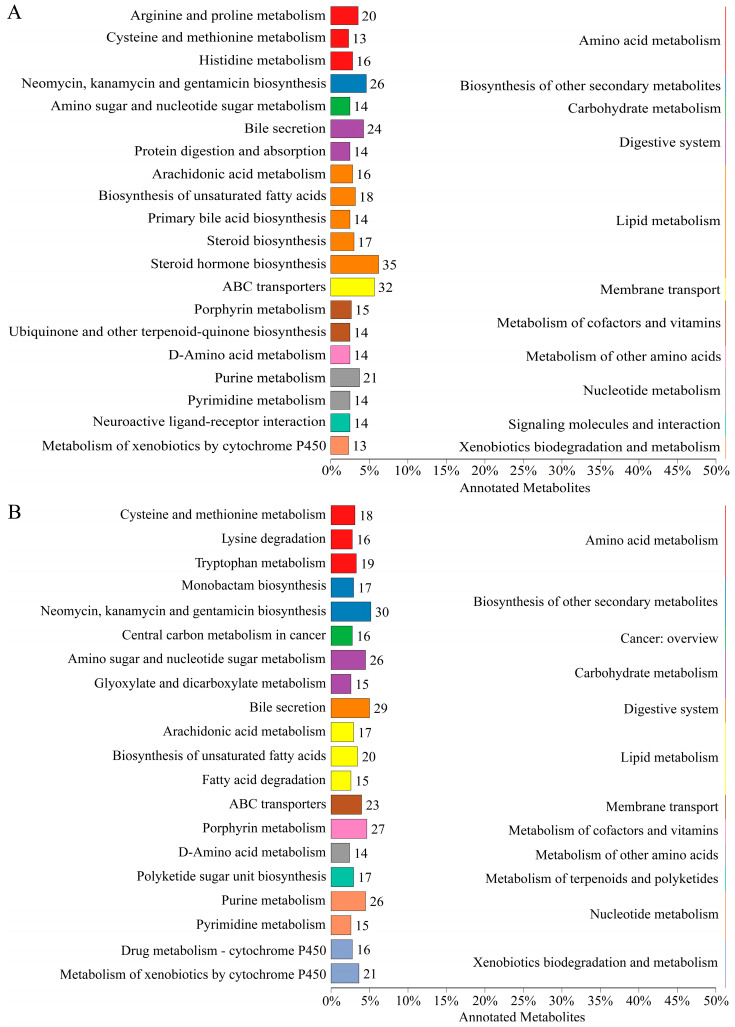
Bar plots illustrate KEGG pathway annotation, showing the number of metabolites mapped to each pathway in positive ion mode (**A**) and negative ion mode (**B**).

The top 20 pathways containing the highest number of metabolites are shown in Figure 3A,B, in positive ion mode and negative ion mode, respectively. Overall, lipids and lipid-like molecules constituted the largest proportion of detected metabolites in both ion modes, followed by organic acids and derivatives.

### 2.3. Multivariate Statistical Analyses of Metabolomic Profiles

**Figure 4:**
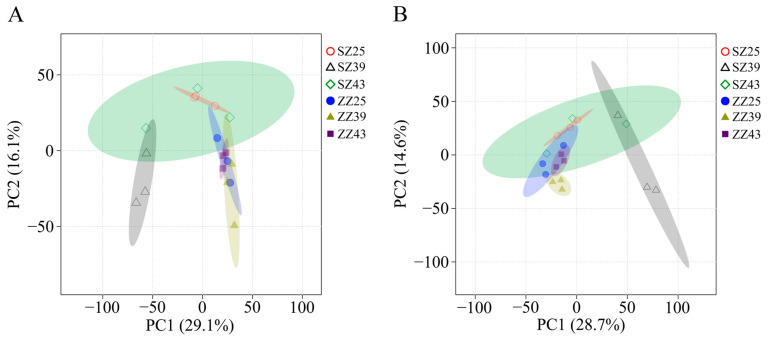
PCA plots of the first two components from six sample groups in positive ion mode (**A**) and negative ion mode (**B**). Note: SZ25 indicates the SZ population treatment group at 25 °C; SZ39 indicates the SZ population treatment group at 39 °C; SZ43 indicates the SZ population treatment group at 43 °C; ZZ25 indicates the ZZ population treatment group at 25 °C; ZZ39 indicates the ZZ population treatment group at 39 °C; ZZ43 indicates the ZZ population treatment group −at 43 °C.

PCA was performed to capture the major variance in the dataset. In positive ion mode, the first principal component (PC1) and the second principal component (PC2) accounted for 29.1% and 16.1% of the total variance, respectively. However, PCA score plots failed to effectively distinguish among the six sample groups (Figure 4A). A similar separation pattern was observed in negative ion mode, where PC1 and PC2 explained 28.7% and 14.6% of the total variance, respectively (Figure 4B).

To further resolve group separations, an orthogonal partial least squares discriminant analysis (OPLS-DA) was conducted. OPLS-DA models demonstrated clear intergroup separation across all comparisons (Figures S1–S8). The model’s predictive performance was assessed using the R2Y and Q2 parameters. All models had R2Y values exceeding 0.992, while most Q2 values ranged between 0.679 and 0.727, confirming model robustness (Table 1). The VIP values obtained from these models were considered reliable for identifying DEMs.

### 2.4. Identification of DEMs

**Figure 5:**
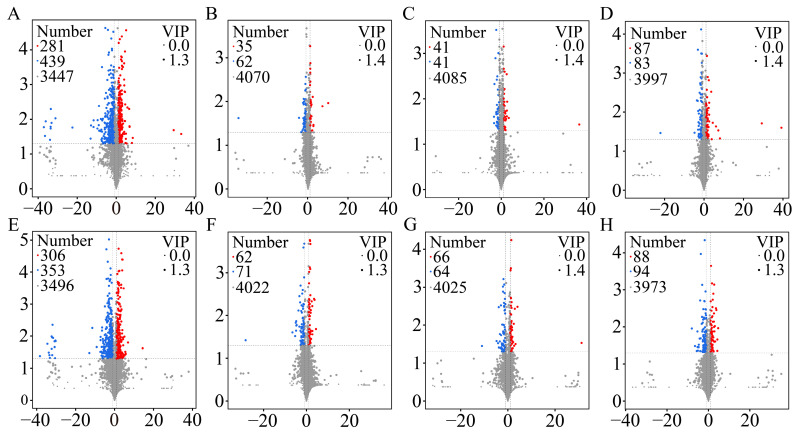
Volcano plots of DEMs in positive ion mode for SZ25 vs. SZ39 (**A**), SZ25 vs. SZ43 (**B**), ZZ25 vs. ZZ39 (**C**), and ZZ25 vs. ZZ43 (**D**) and in negative ion mode for SZ25 vs. SZ39 (**E**), SZ25 vs. SZ43 (**F**), ZZ25 vs. ZZ39 (**G**), and ZZ25 vs. ZZ43 (**H**). The x-axis represents the log2fold change, while the y-axis represents the -log10 *p*-value. Each point corresponds to a metabolite, with the size indicating the VIP value in the OPLS-DA model. Red and blue points represent up-regulated and down-regulated DEMs, respectively, while gray points indicate non-significant metabolites. Horizontal dashed line represents the *p*-value significance threshold (*p*-value < 0.05). Vertical dashed lines indicate the minimum fold change cutoff (|log2foldchange| > 1).

The volcano plots provided a visual representation of the overall expression patterns of DEMs. Metabolites were screened based on three criteria: VIP > 1, *p*-value < 0.05, and |log2foldchange| > 1. The analysis identified 720 DEMs (281 up-regulated and 439 down-regulated) in SZ25 vs. SZ39 (Figure 5A), 97 DEMs (35 up-regulated and 62 down-regulated) in SZ25 vs. SZ43 (Figure 5B), 82 DEMs (41 up-regulated and 41 down-regulated) in ZZ25 vs. ZZ39 (Figure 5C), and 170 DEMs (87 up-regulated and 83 down-regulated) in ZZ25 vs. ZZ43 in positive ion mode (Figure 5D). Notably, the number of DEMs in SZ25 vs. SZ39 was significantly higher than in the other three comparisons. In negative ion mode, 659 DEMs (306 up-regulated and 353 down-regulated) were detected in SZ25 vs. SZ39 (Figure 5E), 133 DEMs (62 up-regulated and 71 down-regulated) in SZ25 vs. SZ43 (Figure 5F), 130 DEMs (66 up-regulated and 64 down-regulated) in ZZ25 vs. ZZ39 (Figure 5G), and 182 DEMs (88 up-regulated and 94 down-regulated) in ZZ25 vs. ZZ43 (Figure 4B and Figure 5H).

**Figure 6:**
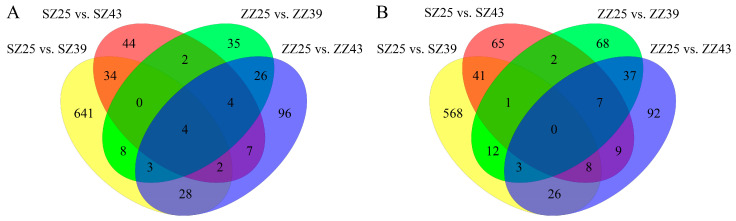
Venn diagrams illustrating unique and shared DEMs across the four comparisons in positive ion mode (**A**) and negative ion mode (**B**).

To further refine the candidate DEMs, Venn analysis was performed, revealing 641, 44, 35, and 96 unique DEMs in SZ25 vs. SZ39, SZ25 vs. SZ43, ZZ25 vs. ZZ39, and ZZ25 vs. ZZ43, respectively, in positive ion mode. Meanwhile, the four DEMs (galabiosylceramide (d18:1/20:0), Lys Leu Ile, cholic acid (may be molecules or sterol derivatives that are structurally or functionally similar to bile acids) and 5′-benzoylphosphoadenosine) are shared across four comparison groups (Figure 6A). It is indicated that the shared differential metabolites likely form an interconnected network that supports thermotolerance; galabiosylceramide stabilizes membrane topology; Lys-Leu-Ile preserves proteome integrity, and 5′-benzoylphosphoadenosine maintains the energy–redox balance, collectively enabling metabolic flux redistribution towards heat-defense prioritization.

The unique DEMs were 568, 65, 68, and 92 from SZ25 vs. SZ39, SZ25 vs. SZ43, ZZ25 vs. ZZ39, and ZZ25 vs. ZZ43, respectively, in negative ion mode. The SZ25 vs. SZ39 consistently exhibited the highest number of unique DEMs (Figure 6B). In summary, metabolomic changes in the SZ groups were more pronounced than in the ZZ groups, with most DEMs uniquely present in the SZ25 vs. SZ39. This suggests that the SZ population may have evolved a unique metabolic pattern to adapt to high-temperature environments.

### 2.5. Enrichment Analyses of DEMs

**Figure 7:**
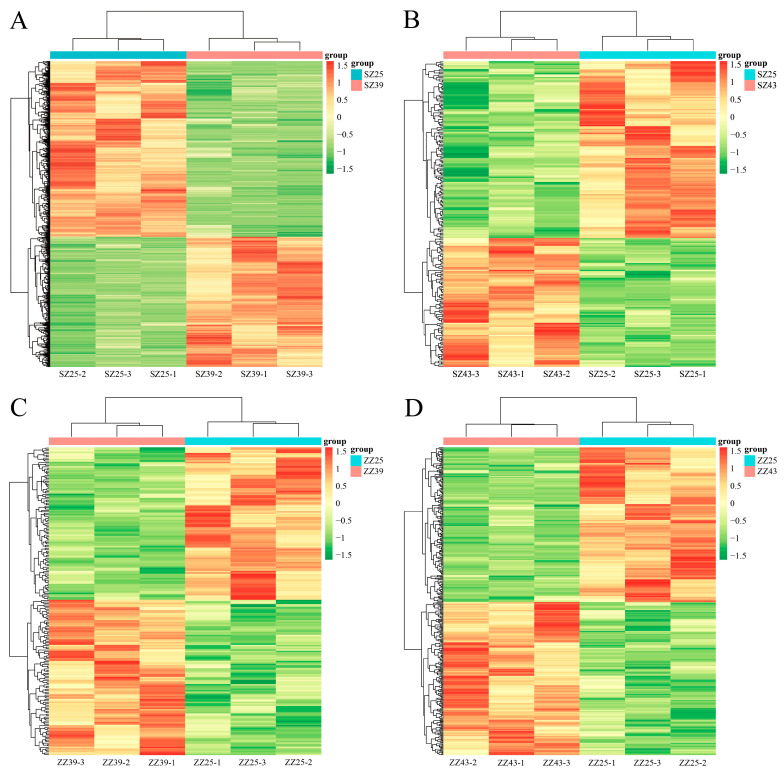
Hierarchical clustering of DEMs in SZ25 vs. SZ39 (**A**), SZ25 vs. SZ43 (**B**), ZZ25 vs. ZZ39 (**C**), and ZZ25 vs. ZZ43 (**D**). The color gradient represents metabolite abundance, ranging from low (green) to high (red).

To further elucidate the metabolic pathways associated with *P. japonica*’s response to AHS, DEMs identified from both positive and negative ion modes within the same comparison were integrated for KEGG enrichment analysis. Hierarchical clustering of DEMs demonstrated clear sample groupings, with biological replicates clustering tightly together in each comparison (Figure 7A–D).

**Figure 8:**
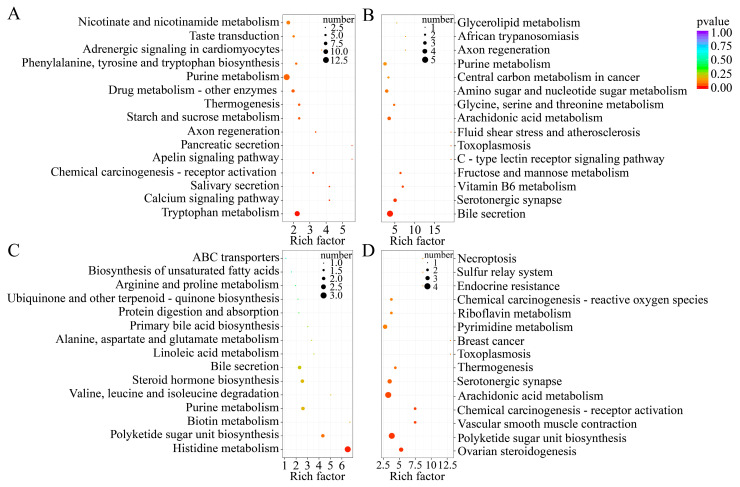
KEGG pathway enrichment analysis for DEMs in SZ25 vs. SZ39 (**A**), SZ25 vs. SZ43 (**B**), ZZ25 vs. ZZ39 (**C**), and ZZ25 vs. ZZ43 (**D**). Pathways with *p*-values < 0.05 are considered significantly enriched. Dot size indicates the number of DEMs within each KEGG pathway, while color intensity represents statistical significance.

In the SZ25 vs. SZ39, 13 DEMs were enriched in purine metabolism, 11 in tryptophan metabolism, and 8 in nicotinate and nicotinamide metabolism. Additionally, two signal transduction pathways, the calcium signaling pathway and the apelin signaling pathway, were significantly enriched (Figure 8A). In the SZ25 vs. SZ43, DEMs were primarily associated with serotonergic synapse (three DEMs) and fructose and mannose metabolism (two DEMs) (Figure 8B). The ZZ25 vs. ZZ39showed the highest enrichment in histidine metabolism (three DEMs) (Figure 8C). Moreover, following AHS exposure at 43 °C for 1 h, notable metabolic changes were observed in ovarian steroidogenesis, polyketide sugar unit biosynthesis, arachidonic acid metabolism, the serotonergic synapse, and pyrimidine metabolism in *P. japonica* from the ZZ region (Figure 8D).

Collectively, purine metabolism (energy homeostasis) and tryptophan-derived serotonergic neurotransmission were the key metabolic pathways across various comparisons under AHS conditions, especially in the SZ population. They predominantly exert critical regulatory functions in energy metabolism, oxidative stress responses, and the dysregulation of neuroendocrine homeostasis, in the context of heat stress responsiveness modulation. These differential metabolites establish a fundamental biochemical substrate underpinning thermal adaptation evolution across geographically divergent *P. japonica* populations.

Ultimately, several metabolic annotations in our dataset (e.g., bile acid biosynthesis and pathways in cancer - specific types) likely stem from technical limitations rather than biological relevance. These include: (1) database biases originating from evolutionarily conserved pathway naming conventions, (2) structural misassignments of insect-derived metabolites, and (3) potential incorporation of microbial metabolites (e.g., bacterial-derived secondary bile acid analogs). Such annotations were carefully contextualized given their physiological incongruence with insect systems.

## 3. Discussion

*P. japonica* has been widely utilized as a biological control agent due to its strong resistance to starvation, high reproductive capacity, exceptional predation efficiency against various agricultural pests, and broad distribution across China [10,11,12,14,16]. Additionally, *P. japonica* exhibits remarkable thermotolerance [13] and has recently expanded its range [16,26]. Given the rising global temperatures due to climate change, this species holds significant potential for further development and application. Therefore, understanding the molecular mechanisms underlying its thermotolerance is of great scientific and practical importance.

Our metabolomic analysis revealed that lipids, lipid-like molecules, organic acids, and their derivatives were the predominant metabolite categories in both positive and negative ion modes. Notably, the metabolic profiles of *P. japonica* varied across geographical populations when subjected to AHS at 39 °C and 43 °C. The number of DEMs identified in the SZ25 vs. SZ39, SZ25 vs. SZ43, ZZ25 vs. ZZ39, and ZZ25 vs. ZZ43 were 1379, 230, 212, and 352, respectively. The significantly higher number of DEMs in SZ39 indicated that *P. japonica* from the SZ region exhibited greater thermotolerance under AHS at 39 °C. Previous studies have shown a significant decline in the survival rate of *P. japonica* adults following exposure to 43 °C for 1 h [18], suggesting that this temperature imposes severe physiological stress. This finding aligns with our results, as the number of DEMs in the SZ25 vs. SZ43 was markedly lower than in the SZ25 vs. SZ39.

KEGG pathway enrichment analysis identified 20 significantly enriched pathways out of 190, with particular emphasis on energy-related metabolic pathways. This aligns with the well-established concept that insect heat tolerance is energy-intensive [27]. Five pathways were of particular interest: two amino acid metabolism pathways (tryptophan and histidine), two carbohydrate metabolism pathways (starch and sucrose and fructose and mannose), and one lipid metabolism pathway (arachidonic acid).

Tryptophan, an essential amino acid, is a precursor to several biologically active compounds, including nicotinic acid (an enzyme cofactor), melatonin (a hormone), and serotonin (a neurotransmitter) [28]. In this study, tryptophan metabolism was the most enriched pathway in the SZ25 vs. SZ39, containing 11 DEMs. Notably, _L_-tryptophan and *N*-formylkynurenine exhibited 6.2-fold and 3.8-fold increases, respectively. High-temperature stress is known to accelerate reactive oxygen species (ROS) accumulation [29,30], and ROS can interact with tryptophan to generate radicals and intermediates, which subsequently rearrange into *N*-formylkynurenine [31], consistent with our findings. Additionally, 3-hydroxy-_L_-kynurenine, an intermediate in the kynurenine pathway, is known to induce neuronal apoptosis at micromolar concentrations [32] and showed a fourfold decrease in our study, suggesting a potential protective response against heat stress.

Histidine plays a fundamental role in protein synthesis and was the most significantly enriched pathway in the ZZ25 vs. ZZ39. The upregulation of D-erythro-1-(imidazol-4-yl)glycerol 3-phosphate, a key metabolite in histidine biosynthesis, was observed. Previous studies have reported that histidine biosynthesis in the brown planthopper (*Nilaparvata lugens*) is mediated by the symbiotic bacterium *Entomomyces delphacidicola* [33]. Furthermore, *Pseudomonas* species and *Streptomyces coelicolor*, which are known to harbor *N*-formimino-_L_-glutamate iminohydrolase (HutF), catalyze the conversion of *N*-formimino-_L_-glutamate to *N*-formyl-_L_-glutamate [34]. In this study, *N*-formimino-_L_-glutamate was significantly downregulated, while *N*-formyl-_L_-glutamate was upregulated in the histidine degradation pathway. Given that *Pseudomonas* was one of the dominant genera identified among the symbiotic bacteria in newly hatched *P. japonica* larvae [35], we hypothesize that symbiotic bacteria may play a critical role in the heat stress response of *P. japonica*.

Two signal transduction pathways, the calcium signaling and apelin signaling pathways, were significantly enriched in the SZ25 vs. SZ39. Calcium ions (Ca^2+^) are universal signaling molecules across cellular processes in prokaryotes and eukaryotes [36]. There is growing evidence of complex interactions between ROS and Ca^2+^, wherein intracellular Ca^2+^ can regulate ROS clearance by activating catalase (CAT) and glutathione (GSH) while increasing superoxide dismutase (SOD) levels, whereas ROS can, in turn, promote Ca^2+^ efflux [37]. Heat stress in *Hu* sheep has been shown to induce ROS accumulation and significant enrichment of the calcium signaling pathway [38]. Thus, calcium signaling may be crucial in heat resistance in *P. japonica*. Apelin, an endogenous peptide, acts as a ligand for the apelin receptor (APJ), which is key in mitigating oxidative stress in the nervous system [39]. Apelin-activated APJ couples with Gq/11 proteins, leading to the generation of inositol trisphosphate (IP3) and the subsequent release of intracellular Ca^2+^ [40,41], suggesting potential interactions between the apelin and calcium signaling pathways. Furthermore, apelin signaling is closely linked to lipid metabolism [42], reinforcing its significance in thermotolerance. The two pathways may synergistically activate enzymes such as SOD and CAT to alleviate oxidative damage, while the calcium signaling pathway could counteract thermal stress by activating heat shock proteins (HSPs), thereby assisting in the repair of misfolded proteins and maintaining cellular homeostasis.

Despite insects being the most diverse organisms on earth, their metabolic responses to heat stress remain poorly understood. In this study, we comprehensively analyzed the metabolomic profile of *P. japonica* under AHS, employing stringent measures to minimize confounding factors, including controlled food supplementation and starvation protocols before AHS exposure. However, certain limitations remain. For instance, previous studies suggest that male and female *P. japonica* adults may exhibit differential thermotolerance when exposed to 43 °C for 1 h [18]. As our study did not account for sex ratios within the samples, potential sex-dependent differences in thermotolerance could have influenced the results. Additionally, this study was conducted under controlled laboratory conditions, which may not fully reflect the complex environmental challenges faced by *P. japonica* in natural habitats.

## 4. Materials and Methods

### 4.1. Specimen Collection and Preparation

Adult *P. japonica* specimens were collected from ZZ, Henan Province (113°35′24″ E, 34°51′36″ N; the average annual temperature, the average temperature of the hottest month, the extreme maximum temperature of the hottest month, and the average minimum temperature of the coldest month are 14.4 °C, 27.5 °C, 43 °C, and −2.1 °C, respectively), and SZ, Guangdong Province, China (113°56′24″ E, 22°48′0″ N; the average annual temperature, the average temperature of the hottest month, the extreme maximum temperature of the hottest month and the average minimum temperature of the coldest month are 22.4 °C, 28.9 °C, 38.7 °C, and 12 °C, respectively). The insects were transported to Henan Agricultural University (113°49′12″ E, 34°48′0″ N) and maintained in a PRX-280C-LED climatic chamber (Ningbo Kesheng Laboratory Instrument, Ningbo, China) at 25 ± 1 °C with a relative humidity of 60 ± 5% and a photoperiod of 16:8 (L:D) h. They were fed *Rhopalosiphum padi* aphids raised on wheat seedlings.

For this experiment, two treatment temperatures (39 °C and 43 °C) were applied to each geographical population, with 25 °C serving as the control. This resulted in six experimental groups: ZZ25, ZZ39, ZZ43, SZ25, SZ39, and SZ43. Each group consisted of three biological replicates, with six *P. japonica* adults per replicate. The experimental procedures were as follows: First, newly emerged adults were individually collected and fed aphids in 25 mL polypropylene cups for three days. To minimize the potential influence of food on metabolomic detection, the adults underwent a 48 h starvation period before AHS treatment. Next, 5-day-old adults were individually placed in 2 mL cryogenic vials, which were then immersed in a preheated water bath at 39 °C or 43 °C for 1 h. Surviving *P. japonica* adults were subsequently cleaned with 75% ethanol, flash-frozen in liquid nitrogen, and stored at −80 °C until metabolite extraction.

### 4.2. Metabolites Extraction

Metabolite extraction followed a standardized protocol. *P. japonica* samples (20 mg) were mixed with 1 mL of an extraction solution containing 40% (*v*/*v*) methanol, 40% (*v*/*v*) acetonitrile, and 20% (*v*/*v*) H_2_O, then vortexed for 30 s. The samples were homogenized using a 45 Hz grinder for 10 min, followed by sonication in an ice-water bath for 10 min. After incubation at −20 °C for 1 h, the samples were centrifuged at 12,000× *g* for 15 min at 4 °C.

To redissolve the dried extract from 500 μL of supernatant, 160 μL of a secondary extraction solution (50% (*v*/*v*) acetonitrile and 50% (*v*/*v*) H_2_O) was added, followed by vortexing for 30 s, sonication in an ice-water bath for 10 min, and centrifugation at 12,000× *g* for 15 min at 4 °C. Finally, 120 μL of the supernatant was transferred into 2 mL LC-MS vials for metabolite analysis. Quality control (QC) samples were prepared by pooling 10 μL of supernatant from each sample.

### 4.3. UPLC-MS/MS Analysis

Metabolomic profiling was performed using an ACQUITY I-Class PLUS ultra-high-performance liquid chromatography system (Waters Corporation, Milford, MA, USA) coupled with a Xevo G2-XS QTof high-resolution mass spectrometer (Waters Corporation, Milford, MA, USA) equipped with an electrospray ionization (ESI) source. The ESI source parameters were as follows: capillary voltage (2000 V in positive mode and −1500 V in negative mode), cone voltage (30 V), ion source temperature (150 °C), desolvation gas temperature (500 °C), backflush gas flow rate (50 L/h), and desolvation gas flow rate (800 L/h). The mass spectrometer operated in MSe mode using the MassLynx software (V4.2, Waters Corp., Milford, CT, USA), scanning within an *m*/*z* range of 50–1200. Collision energies were set at 2 V for low-energy scans and 10–40 V for high-energy scans, with a scan frequency of 0.2 s.

Chromatographic separation was conducted using an ACQUITY UPLC HSS T3 column (2.1 mm × 100 mm, 1.8 μm; Waters Corporation). The mobile phase consisted of 0.1% formic acid in H_2_O (solvent A) and 0.1% formic acid in acetonitrile (solvent B). The elution gradient was as follows: 98% A (2% B) for 0.25 min, transitioning to 2% A (98% B) over 9.75 min, held for 3 min, then returning to 98% A (2% B) over 0.1 min, and maintained for 1.9 min. The flow rate was set at 400 μL/min, with an injection volume of 1 μL per sample. Each sample was analyzed twice, once in positive ion mode and once in negative ion mode.

### 4.4. Raw Data Preprocessing

Raw data were processed using the Progenesis QI software (Waters Corp., Milford, CT, USA) for peak extraction and alignment. Metabolite quantification and theoretical fragment identification were conducted using the METLIN database (Waters Corp., Milford, CT, USA) and a self-compiled reference database. Theoretical fragment matching was performed with a mass deviation threshold of 100 ppm to ensure high-accuracy identification.

### 4.5. Statistical Analyses

Both univariate and multivariate statistical analyses were performed. Principal Component Analysis (PCA) was conducted using MetaboAnalyst 6.0 (https://www.metaboanalyst.ca, accessed on 8 November 2024) to evaluate intra-group sample consistency and inter-group variations. Metabolite abundance data were normalized via autoscaling (mean centered and divided by the standard deviation of each variable) and log transformation. Orthogonal partial least squares discriminant analysis (OPLS-DA) was implemented using the “ropls” R package 1.6.2 to distinguish between sample groups and identify key metabolites based on the variable importance in projection (VIP) scores, calculated via multiple cross-validation. The reliability of OPLS-DA models was confirmed through 200 permutation tests.

Student’s *t*-tests were conducted to assess the statistical significance of metabolite differences between groups, with *p*-values used to determine significance thresholds. Foldchange values were calculated as the ratio of mean metabolite abundance in the treated versus control conditions. Differentially expressed metabolites (DEMs) were subjected to KEGG pathway enrichment analysis via the BMK Cloud platform (https://www.biocloud.net, accessed on 20 November 2024) to identify metabolic pathways associated with AHS. Venn diagrams were generated to visualize common and unique DEMs across comparisons, while heatmaps were constructed using the “pheatmap” R package 1.0.2 with unit scaling.

### 4.6. Metabolite Annotation and DEM Screening

Metabolites were annotated using the Human Metabolome Database (HMDB, https://hmdb.ca, accessed on 5 May 2024), the Kyoto Encyclopedia of Genes and Genomes (KEGG, https://www.genome.jp/kegg, accessed on 5 May 2024), and the LIPID MAPS Structure Database (LMSD, https://lipidmaps.org, accessed on 5 May 2024) (Tables S3–S12). DEMs were identified based on the following criteria: VIP > 1, *p*-value < 0.05, and |log2foldchange| > 1.

Some annotations (e.g., bile acid biosynthesis) may reflect database biases, as KEGG pathways are primarily curated from mammalian systems and lack insect-specific metabolic references.

## 5. Conclusions

Temperature is a key factor influencing species performance [43], but extreme heat from intensified global warming can induce heat stress in animals. Using untargeted UPLC-MS/MS-based metabolomics, we revealed that lipids, organic acids, and energy-related metabolic pathways, including tryptophan, histidine, starch and sucrose, arachidonic acid, and fructose and mannose metabolism, play crucial roles in the AHS response of *P. japonica* from two geographical populations. These components interact synergistically: lipids stabilize membranes to protect metabolic enzymes; organic acids maintain the tricarboxylic acid (TCA) cycle flux and redox balance; energy pathways fuel repair mechanisms. This integrated system allows *P. japonica* to survive in extreme temperatures by balancing structural integrity, metabolic flexibility, antioxidant defense, and energy allocation. In general, the SZ population experiences more frequent heat stress than the ZZ population due to geographical differences. This is consistent with the significant activation of the purine and tryptophan metabolic pathways in the SZ population, which indicated that these pathways mediate thermal adaptation via coordinated energy reprogramming, enhanced antioxidant defenses, and neuroendocrine homeostasis regulation. The deciphering of these metabolic networks provides a critical entry point for research on ecological adaptation in the context of climate change and contributes to the breeding of heat-tolerant strains in natural enemy insects.

## Figures and Tables

**Table 1:** Parameters of established OPLS-DA models in positive ion mode (bold font) and negative ion mode (regular font).

Comparisons	R2Y	Q2
SZ25 vs. SZ39	**0.998**	**0.942**
	0.997	0.941
SZ25 vs. SZ43	**0.992**	**0.68**
	1	0.72
ZZ25 vs. ZZ39	**0.999**	**0.693**
	0.999	0.679
ZZ25 vs. ZZ43	**0.999**	**0.714**
	0.999	0.727

## Data Availability

Data are contained within the article and Supplementary Materials.

## Supplementary Materials

The following supporting information can be downloaded at https://www.mdpi.com/article/10.3390/ijms26104541/s1.
