# Supplementary material for: Changes in Metabolomics Profiles of Propylea japonica in Response to Acute Heat Stress
Source: Int J Mol Sci. 2025 May 9;26(10):4541. doi: 10.3390/ijms26104541 (PMC12110978; doi:10.3390/ijms26104541)
Supplement: Supplementary file 1 [file ijms-26-04541-s001.zip › Figure S1-S8.pdf]

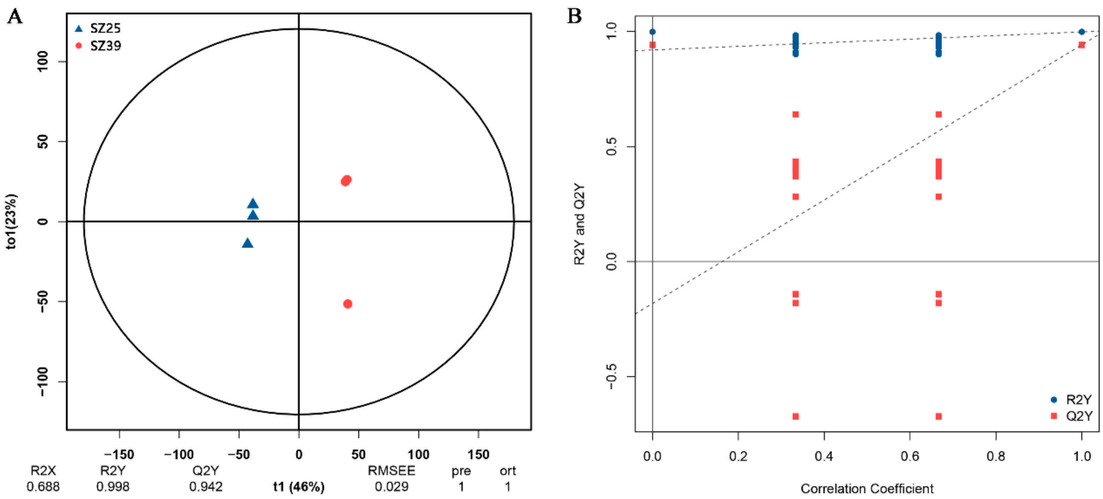

Supplementary Figure S1. OPLS-DA scores **(A)** and permutation test **(B)** plots of SZ25 vs. SZ39 comparison in positive ion mode.

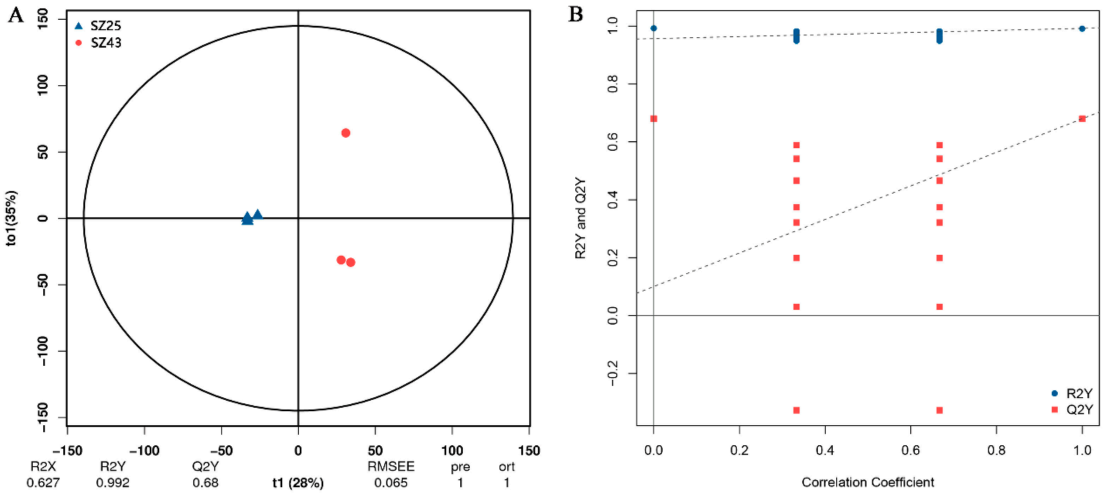

Supplementary Figure S2. OPLS-DA scores **(A)** and permutation test **(B)** plots of SZ25 vs. SZ43 comparison in positive ion mode.

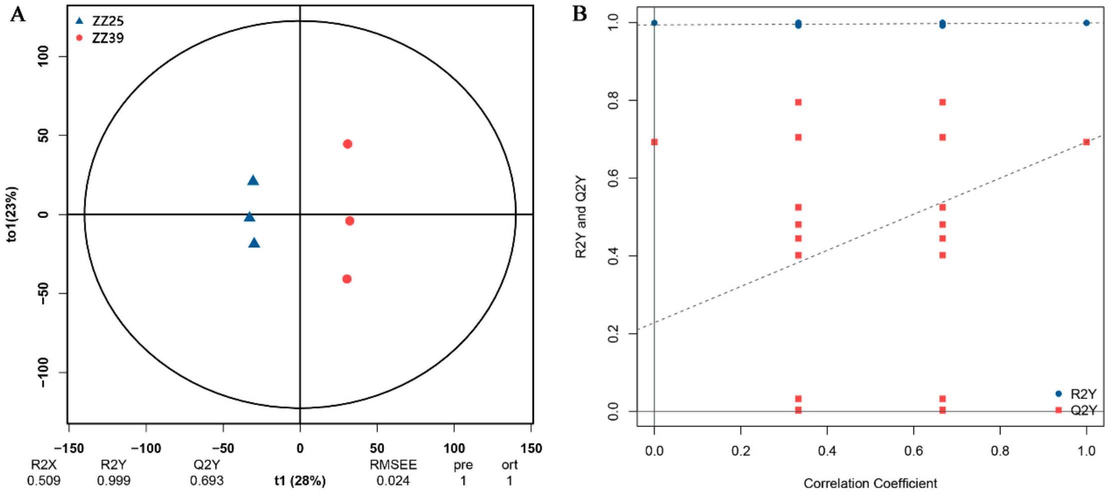

Supplementary Figure S3. OPLS-DA scores **(A)** and permutation test **(B)** plots of ZZ25 vs. ZZ39 comparison in positive ion mode.

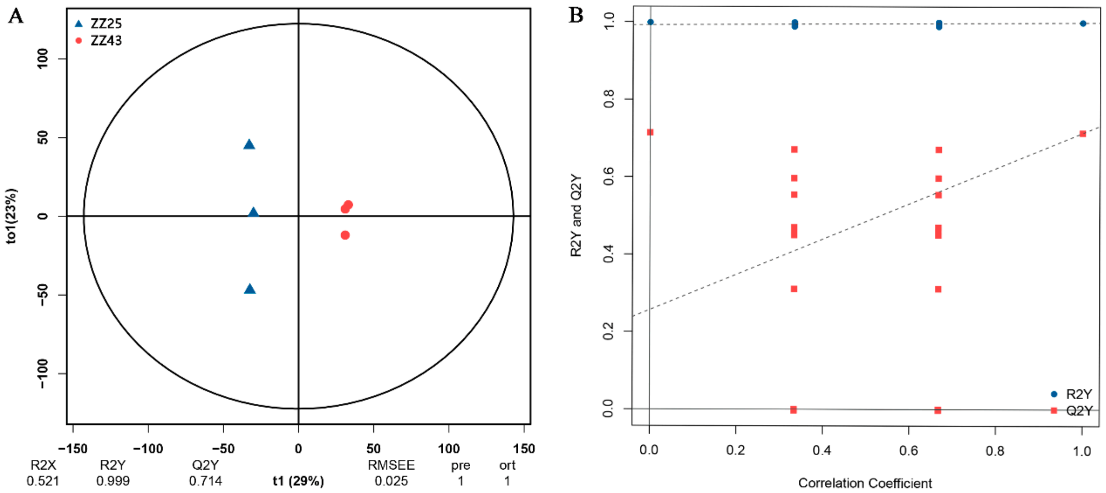

Supplementary Figure S4. OPLS-DA scores **(A)** and permutation test **(B)** plots of ZZ25 vs. ZZ43 comparison in positive ion mode.

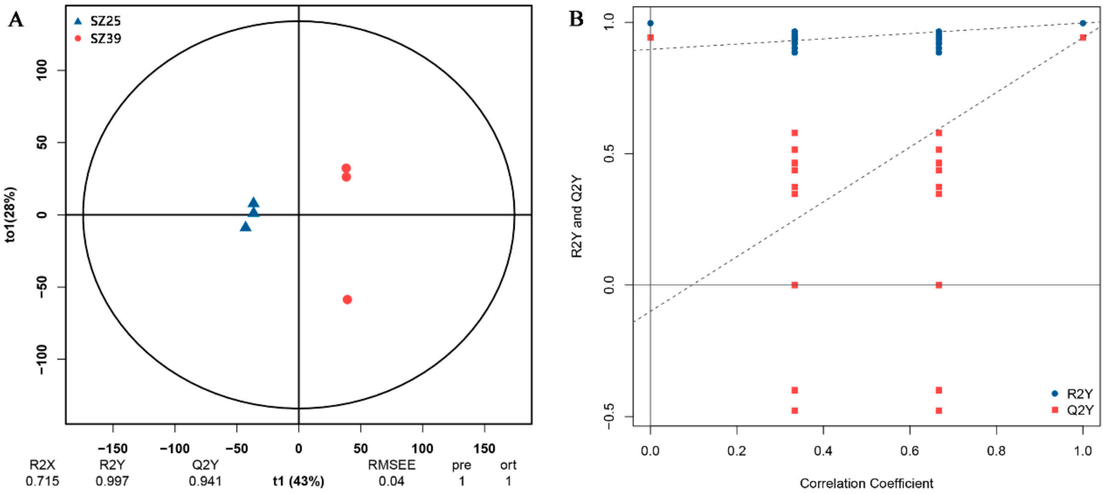

Supplementary Figure S5. OPLS-DA scores **(A)** and permutation test **(B)** plots of SZ25 vs. SZ39 comparison in negative ion mode.

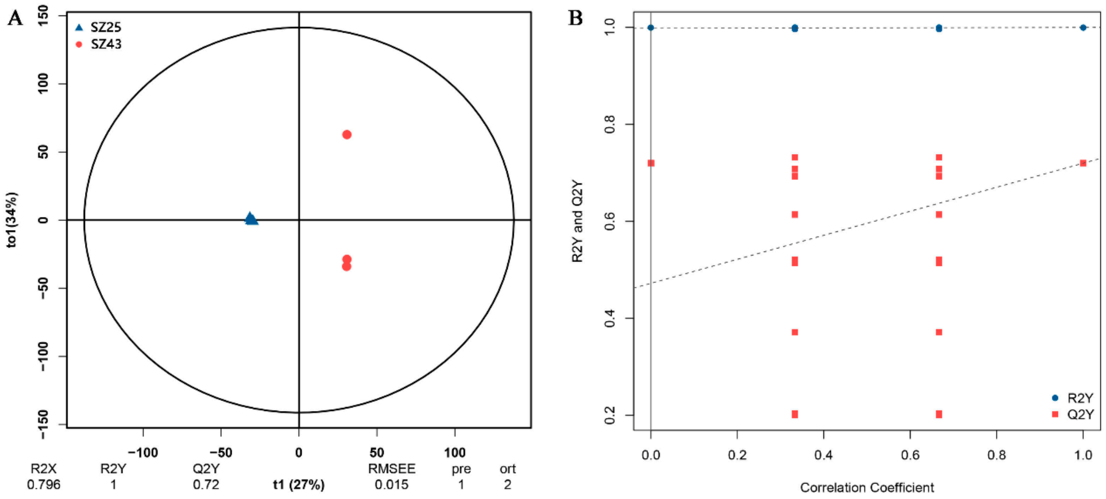

Supplementary Figure S6. OPLS-DA scores **(A)** and permutation test **(B)** plots of SZ25 vs. SZ43 comparison in negative ion mode.

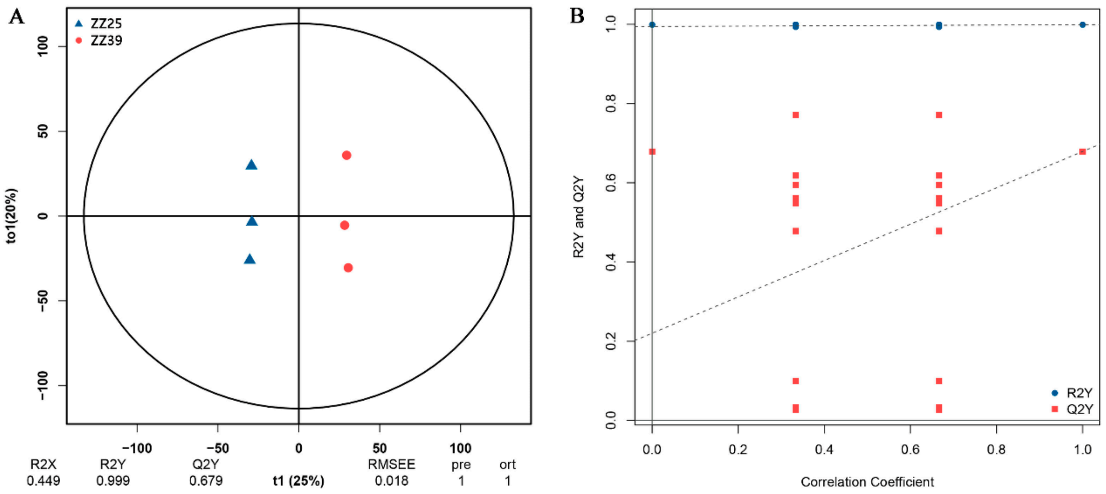

Supplementary Figure S7. OPLS-DA scores **(A)** and permutation test **(B)** plots of ZZ25 vs. ZZ39 comparison in negative ion mode.

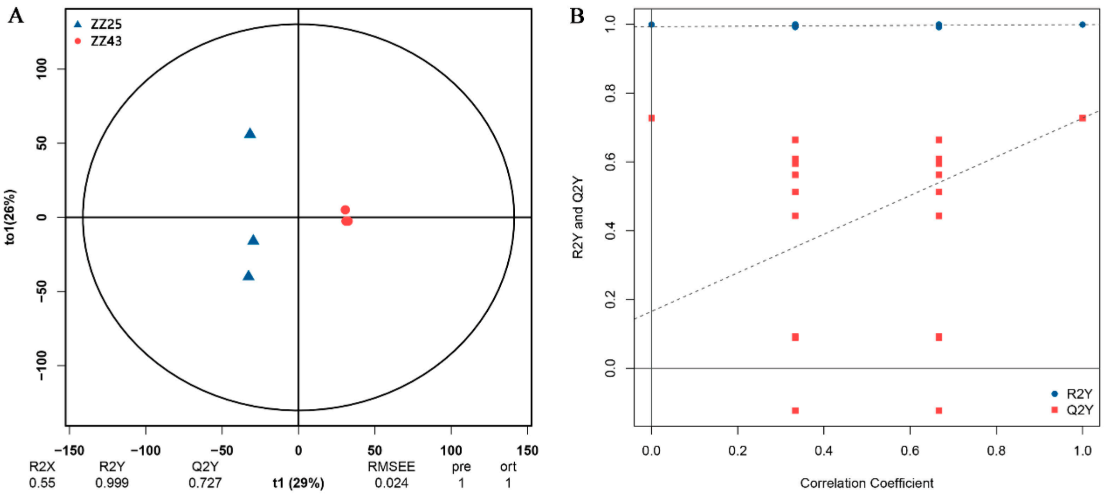

Supplementary Figure S8. OPLS-DA scores **(A)** and permutation test **(B)** plots of ZZ25 vs. ZZ43 comparison in negative ion mode.
